# Association between Albuminuria and Different Body Constitution in Type 2 Diabetes Patients: Taichung Diabetic Body Constitution Study

**DOI:** 10.1155/2015/603048

**Published:** 2015-10-26

**Authors:** Cheng-Hung Lee, Tsai-Chung Li, Chia-I Tsai, Shih-Yi Lin, I-Te Lee, Hsin-Jung Lee, Ya-Chi Wu, Yi-Chang Su

**Affiliations:** ^1^Graduate Institute of Chinese Medicine, College of Chinese Medicine, China Medical University, Taichung 40402, Taiwan; ^2^Department of Traditional Chinese Medicine, Han Ming Hospital, Changhua 50072, Taiwan; ^3^School of Chinese Medicine, College of Chinese Medicine, China Medical University, Taichung 40402, Taiwan; ^4^Graduate Institute of Biostatistics, China Medical University, Taichung 40402, Taiwan; ^5^Department of Health Administration, College of Health Science, Asian University, Taichung 41354, Taiwan; ^6^Department of Traditional Chinese Medicine, Taichung Veterans General Hospital, Taichung 40705, Taiwan; ^7^Division of Endocrinology and Metabolism, Department of Internal Medicine, Taichung Veterans General Hospital, Taichung 40705, Taiwan; ^8^Institute of Medicine, Chung Shan Medical University, Taichung 40201, Taiwan; ^9^School of Medicine, National Yang-Ming University, Taipei 11221, Taiwan; ^10^Division of New Drugs, Center for Drug Evaluation, Taipei 11557, Taiwan

## Abstract

*Objective*. Albuminuria in type 2 diabetes mellitus (T2DM) patients increases the risk of diabetic nephropathy, the leading cause of end-stage renal disease worldwide. Because albuminuria is modifiable, identifying relevant risk factors could facilitate prevention and/or management. This cross-sectional study investigated whether body constitution (BC) independently predicts albuminuria. *Method*. Patients with T2DM (*n* = 846) received urinalysis, a blood test, and diabetic retinopathy examination. Albuminuria was defined by an elevated urinary albumin/creatinine ratio (≥30 *μ*g/mg). BC type (Yang deficiency, Yin deficiency, and Phlegm stasis) was assessed using a body constitution questionnaire (BCQ). Traditional risk factors for albuminuria were also recorded. Odds ratios (ORs) of albuminuria for BC were estimated using multivariate logistic regression. *Results*. Albuminuria was more prevalent in patients with Yang deficiency or Phlegm stasis (both *P* < 0.01). After adjustment, patients with both Yang deficiency and Phlegm stasis exhibited a significantly higher risk of albuminuria (OR = 3.037; 95% confidence interval = 1.572–5.867, and *P* < 0.001). *Conclusion*. BC is strongly associated with albuminuria in T2DM patients. Using a BCQ to assess BC is noninvasive, convenient, and inexpensive and can provide information for health care professionals to identify T2DM patients who are at a high risk of albuminuria.

## 1. Introduction

Global prevalence of diabetes, a chronic metabolic disease, has increased rapidly and is estimated to reach over 552 million by 2030 [1]. Diabetic nephropathy, a severe vascular complication of diabetes, is the leading cause of end-stage renal disease (ESRD) in many countries [2, 3]. ESRD considerably influences public health and health care economy [4–6]. According to the annual report of the United States Renal Data System (USRDS), Taiwan had the world's highest incidence and prevalence of ESRD during 2002–2005 and 2009, respectively [4, 7]. The Taiwan Society of Nephrology demonstrated that the increasing prevalence of diabetes was the main cause of the rising prevalence and incidence of ESRD in Taiwan [8]. Albuminuria is a modifiable and crucial risk factor for diabetic nephropathy [9, 10]. In addition, multinational and regional studies have revealed that Asian diabetic populations have a higher prevalence of albuminuria [11, 12]. Hence, reducing the risk of albuminuria is a key treatment goal for renal protection in patients with type 2 diabetes (T2DM) to prevent the progression of diabetic nephropathy.

Despite the vast efforts devoted to managing the potential risk factors for albuminuria, the global incidence of ESRD in patient with T2DM continues to rise [5, 8, 13]. This is probably because the pathogenesis of albuminuria is multifactorial, thus indicating an urgent necessity to discover other potential risk factors. Traditional Chinese medicine (TCM) may provide a novel insight into this problem. TCM, a type of frequently used complementary and alternative medicine (CAM) [14–16], emphasizes the concept of personalized medicine based on body constitution (BC) theory [17–19]. An individual's constitution status is formed by the state of Yang and Yin in his body. Yin and Yang deficiency BCs refer to the decrease of the material and energy level, respectively, and the imbalance between Yin and Yang may cause Phlegm stasis [17]. People with different BC types are variously prone to certain diseases and differ in disease progression [20, 21], and TCM practitioners treat patients with the same disease diagnosis differently according to each individual's body constitution, which is known as* tong bing yi zhi* in Chinese. Besides, to achieve optimal health promotion, TCM practitioners used to adopt individualized preventive methods based on BC [22–24].

Distinguishing T2DM patients who have a higher risk of albuminuria is essential for prevention or early treatment of diabetic nephropathy. In the current study, we sought to determine whether BC could be an independent predictor of albuminuria in 846 patients with T2DM recruited from a medical center with information of their BC status and data from urinalysis, blood test, and diabetic retinopathy (DR) examination.

## 2. Materials and Methods

### 2.1. Study Design and Participants

**Figure 1:**
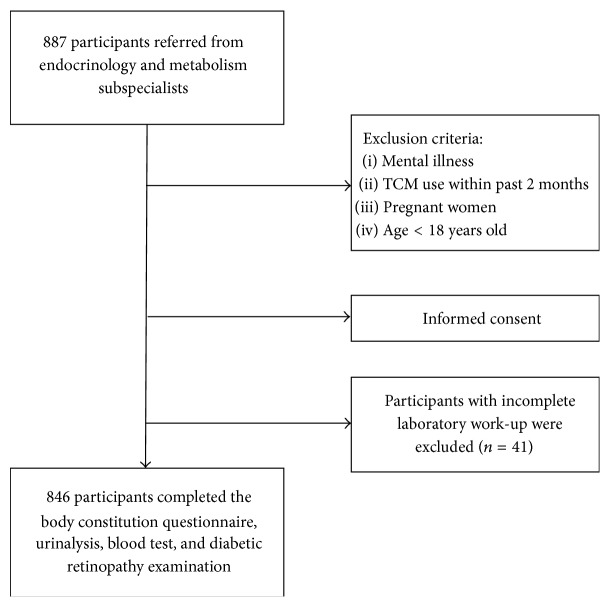
The flowchart of the study.

This cross-sectional study was conducted from February 2010 to February 2011 at the Diabetes Health Promotion Center of Taichung Veterans General Hospital in Taichung, Taiwan. The study protocol was approved by the Institutional Review Board of Taichung Veterans General Hospital (C10007). A total of 887 participants diagnosed with T2DM were referred by endocrinology and metabolism subspecialists from an outpatient clinic. Written informed consent was obtained from each participant. Every participant had to undergo the following tests for determining the risk factors for albuminuria: BC measurement, sociodemographic characteristics (including gender, age, body mass index, and waist circumference), lifestyle behaviors, diabetic history, lipid profile, blood pressure, kidney function, and DR. All the tests were performed on the same day. Forty-one participants who could not complete all laboratory tests were excluded. A total of 846 participants with T2DM were included in the final analysis.  Figure 1 shows the recruitment flowchart of the study participants.

If the sample size is fixed at 800 patients with type 2 diabetes, the power would be 0.8891, given that the association between BC and albuminuria (OR) was 2 with two-sided type 1 error of 5% and prevalence of 12.5% for BC. This is calculated with the use of a two-sided proportion test (*z* test) on the assumption that there is an albuminuria prevalence of 57.1% in patients with type 2 diabetes whose BC was Yang deficiency. This information came from our pilot study and Yang deficiency was the primary predictor of BC for albuminuria in study design stage.

### 2.2. Measurements

#### 2.2.1. Body Constitution Measurement

All the participants were self-administered a body constitution questionnaire (BCQ) to evaluate their BC status. The items of the BCQ were generated from TCM textbooks and the published literature [17, 21, 25]. The initial items were translated into colloquial questions through a 2-stage Delphi process. The resulting questionnaire was tested to check for wording, sequencing, grammar, and ease of comprehension. Then, intraclass consistency was done to reduce the items of the questionnaire [17, 21, 25]. The BCQ demonstrates favorable factorial validity [21], and the Cronbach *α* of each constitution subscale in previous studies has been between 0.88 and 0.90 [21, 26, 27]. The BCQ comprised 44 items on a 5-point Likert-type scale from 1 (*never happened*) to 5 (*always happens*), including 19 items on Yang deficiency [17, 26], 16 items on Phlegm stasis [21], and 19 items on Yin deficiency [25, 27]. Some items belonging to these three scales overlapped, and the final score of each constitution was calculated by summing the scores of all items on each subscale. A higher score implied a greater deviation from the constitution. The score range of Yang deficiency is between 19 and 95, and the participant was diagnosed with Yang deficiency when the score reached over 30.5 [26]. For Phlegm stasis, the score range is 16 to 80, and the cut point for diagnosis is 26.5 [21]. As for Yin deficiency, the score range is 19 to 95, and the participant was diagnosed with Yin deficiency BC when the score is higher than 29.5 [27].

#### 2.2.2. Detection of Albuminuria

Spot urine samples were collected from each participant and the urinary albumin concentrations were measured using immunoturbidimetry [28] at Taichung Veterans General Hospital. Daily urinary albumin secretion was estimated by calculating an elevated urinary albumin/creatinine ratio (ALB/Cr) [29–31]. Albuminuria was defined according to an elevated urinary albumin/creatinine ratio (≥30 *μ*g/mg) [30, 31].

#### 2.2.3. Detection of Diabetic Retinopathy

Each participant received standardized central fundus photographic imaging and both eyes of each participant were photographed using a nonstereoscopic 45° digital nonmydriatic camera (CR-DGi, Canon, Inc., Tokyo, Japan). Experienced and trained endocrinology and metabolism subspecialists examined the fundus photographs in a masked manner. The DR severity of each eye was graded according to the International Clinical Diabetic Retinopathy and Diabetic Macular Edema Disease Severity Scales [32]. Participants who had at least one eye with either nonproliferative DR or proliferative DR were assigned to the DR group.

### 2.3. Data Collection

Traditional risk factors for albuminuria were derived to control for the confounding influence. The sociodemographic characteristics (gender, age, height, and waist circumference), lifestyle behaviors (smoking history, alcohol consumption, and exercise habits), diabetes history (diabetes duration, oral hypoglycemia agent, and insulin usage), and systolic and diastolic blood pressure of all the participants were investigated through personal interviews at the Diabetes Health Promotion Center of Taichung Veterans General Hospital. Fasting (>12 hours) blood samples were collected for measuring the level of fasting blood sugar, glycosylated hemoglobin (HbA1c), total cholesterol, total triglyceride, high-density lipoprotein, low-density lipoprotein (LDL), and creatinine (Cr). The estimated glomerular filtration rate (eGFR) was calculated using the Modification of Diet in Renal Disease four-variable equation: 186 × serum creatinine − 1.154 × age − 0.203 × 1.212 (if black) × 0.742 (if female) [33].

### 2.4. Statistical Analysis

Continuous and categorical variables were presented as mean ± standard deviation (SD) and number (%), respectively. For comparing the differences between groups, chi-square test and *t*-test were used for categorical and continuous variables, respectively.

In the other published paper from Taichung Diabetic Body Constitution Study (TDBS), the independent effects of Yang deficiency, Phlegm stasis, and Yin deficiency on DR among T2DM patients had been explored [34]. In this study, we are interested in albuminuria, another diabetic microvascular complication. In addition to examining independent effects of BCs, we further examined their joint effect of different BCs on albuminuria.

We used hierarchical models for covariant variables to determine whether BC is an independent predictor of albuminuria. First, crude ORs were calculated without adjustment. Subsequently, sociodemographic characteristics, lifestyle behaviors, blood pressure, lipid profile, diabetes history, eGFR, and DR were sequentially entered into the model. Finally, the joint effect of Yang deficiency and Phlegm stasis on albuminuria was examined. A two-sided significance level was set at *P* < 0.05. All analyses were performed using SAS version (SAS Institute Inc., Cary, NC, USA).

## 3. Results

The study group comprised 366 (43%) females and 480 (57%) males with a mean age of 63.72 years (SD = 13.05 years), with a mean duration of diabetes of 8.92 years (SD = 7.92 years). Among the study participants, 232 (27.4%), 112 (13.2%), and 99 (11.7%) were diagnosed with Yin deficiency, Phlegm stasis, and Yang deficiency, respectively.  Table 1 shows a comparison of sociodemographic characteristics, lifestyle behaviors, diabetic history, lipid profile, blood pressure, kidney function, and DR among the participants with and without Yang deficiency, Yin deficiency, and Phlegm stasis. Participants with Yin deficiency had a higher mean age. Patients with Yang deficiency, Phlegm stasis, and Yin deficiency had a higher proportion of females than those without corresponding BC. Patients with Phlegm stasis were less likely to have alcohol consumption and regular exercise habits. These patients had higher BMI and waist circumference. Higher percentage of insulin usage was noted in participants with Yang or Yin deficiency. Participants with Yin deficiency had lower eGFR level. Participants with Yang deficiency or Phlegm stasis were less likely to develop DR.

Among the study participants, 363 (42.9%) showed elevated urine albumin excretion (urinary albumin/creatinine ratio ≥ 30 *μ*g/mg).  Table 2 shows the prevalence of albuminuria according to BC types. Participants with Yang deficiency or Phlegm stasis had significantly higher prevalence of albuminuria (56.57% versus 41.10% and 56.25% versus 40.87%, resp., both *P* < 0.01).

Table 3 lists the unadjusted and hierarchically adjusted ORs for albuminuria associated with each BC type. Participants with Yang deficiency or Phlegm stasis were more likely to develop albuminuria (crude OR = 1.87, 95% CI = 1.22–2.85, 1.860, and 1.25–2.78, resp.). After adjustment for other risk factors, including sociodemographic characteristics, lifestyle behaviors, blood pressure, lipid profile, diabetes history, eGFR, and DR, Yang deficiency and Phlegm stasis remained strongly associated with albuminuria (OR = 2.26, 95% CI = 1.36–3.75, 1.92, and 1.19–3.08, resp.). In addition, significant joint effect of Yang deficiency and Phlegm stasis on albuminuria (OR = 3.037, 95% CI = 1.57–5.87) was observed (Table 4).

## 4. Discussion

In our study, we considered traditional risk factors for albuminuria, including HbA1c, systolic blood pressure, DR, duration of diabetes, kidney function, and smoking [11]. After multivariate adjustment, the results of this cross-sectional study suggest that Yang deficiency and Phlegm stasis were independent risk factors for albuminuria. In addition, a significant joint effect of Yang deficiency and Phlegm stasis on albuminuria was noted. T2DM patients who had both Yang deficiency and Phlegm stasis were three times more likely to develop albuminuria.

Based on our research, this is the first clinical study to evaluate the association between albuminuria and BC in patients with T2DM. People with different BC types are more prone to certain diseases than others [17, 20, 35]. According to TCM theory, a person's BC is formed by Yin and Yang, and an imbalance between the two may cause Phlegm stasis. Yang deficiency implies that an energy level responsible for maintaining bodily functions has diminished [17], whereas Yin deficiency implies diminishing of materials (including blood, body fluid, and essence) in performing bodily functions [25]. Phlegm stasis is induced when the materials transported by the energy are impeded by external or environmental stimuli [21].

In TCM, diabetic nephropathy is referred to as an intrinsically deficient but extrinsically excessive syndrome. Deficiency of qi and excess of phlegm stasis are believed to be the main pathologic mechanism responsible for development of diabetic nephropathy [36]. Several clinical trials have aimed to discover the efficacy of TCM on diabetic proteinuria, and the results suggest that Chinese herbal medicine seems to be an effective and safe therapy option [36]. The three most commonly used herbs in different herbal preparations are* Astragalus* (Huang Qi),* Salvia miltiorrhiza* (Dan Shen), and* Poria* (Fuling), consecutively [36]. In TCM, the* Astragalus* has the effect of replenishing Qi [36, 37].* Salvia miltiorrhiza* and* Poria* are used to activate blood circulation and to resolve phlegm [36]. By using epidemiology module, our study results, that T2DM patients with both Yang deficiency and Phlegm stasis are at a threefold risk of exhibiting albuminuria, successfully correspond with the pathology mechanism and clinical usage of certain Chinese medicine herbs.

Health promotion and disease prevention are essential in TCM. BC is modifiable and may transform as time passes or when a crucial health event occurs [38]. An epidemiological study revealed that the factors influencing BC include emotions, body weight, educational level, mental work, age, and exercise habit [39]. Chinese people have endeavored to improve their health for the past thousand years by adjusting their unbalanced BC status. A recent clinical study proved that Chinese food therapy, an effective nonpharmacological approach, can restore the Yin-Yang harmony, improve the quality of life, control blood pressure, and minimize disease symptoms in hypertensive patients with Yin deficiency [40].

From public health perspectives, screening, monitoring, and treating patients with albuminuria are strongly recommended for preventing chronic kidney disease and cardiovascular disease [41]. However, an early detection of albuminuria requires a particular but expensive immunochemical test. The questionnaire, BCQ, with favorable reliability and validity [17, 21, 25–27], has been used to distinguish patients who had different risks of certain diseases [34, 42, 43]. Furthermore, the BCQ facilitates a noninvasive, convenient, fast, and inexpensive method that can be easily applied by health care professionals to assess a patient's BC status. Our results can aid health care professionals in identifying patients with diabetes who are at a high risk of albuminuria.

With the rising burden of chronic illness and global aging population, public health research in integrative and complementary medicine has become essential [44]. People who use CAM have a greater degree of health-seeking behavior to prevent disease and promote health wellness compared with those who do not; thus, CAM providers play a critical role in health promotion and disease prevention [45]. A previous study revealed that general practitioners with more Chinese medicine knowledge referred their patients to TCM practitioners more frequently [46]. Hence, it is crucial to provide scientific evidence in support of CAM or TCM concepts that can aid in disease prevention and health promotion and to share the newly established information with health care providers. Thus, people can integrate health service effectively and safely. Therefore, we launched the Taichung Diabetic Body Constitution Study (TDBS) to evaluate the effect of BC on patients with T2DM [34, 47] and to continue following the study cohort for determining the longitudinal effect.

Our study has three major limitations. First, a potential selection bias may exist because all the study participants were recruited from a medical center. The disease severity of patients with T2DM treated at a medical center may differ from that of patients with T2DM treated in other clinical settings. The participants in our study may have had more comorbidities, poorer control of blood sugar, and a longer duration of diabetes compared with other patients. Nevertheless, the results can be applied to other T2DM patients exhibiting similar disease characteristics. Second, there was a potential confounding effect caused by other unmeasured variables because this was an observational study. We included most of the confounding factors reported in the literature to minimize the possibility of a confounding effect. Finally, we examined a cross-sectional association, which cannot make causal inference because it lacked time sequence. A cohort study is necessary to determine the casual relationship.

## 5. Conclusion

CAM is a public health resource for increasing the prevention of certain disease and promoting health. Distinguishing patients with T2DM who exhibit an increased risk of albuminuria is crucial for preventing diabetic nephropathy. According to TCM theory, BC is modifiable, and different BC types may affect the development and prognosis of certain diseases differently. The results of the current study suggest that T2DM patients who have both Yang deficiency and Phlegm stasis are at a threefold risk of developing albuminuria. Using BCQ to assess BC status is noninvasive, convenient, fast, and inexpensive and should be adopted in clinical practice to target patients with diabetes who are at a high risk of albuminuria.

## Figures and Tables

**Table 1:** Participants' characteristics.

	Yang deficiency (*n* = 846)			Phlegm stasis (*n* = 846)			Yin deficiency (*n* = 846)		
	Yes (*n* = 99)	No (*n* = 747)	*P* value	Yes (*n* = 112)	No (*n* = 734)	*P* value	Yes (*n* = 232)	No (*n* = 614)	*P* value
Age (years)	62.56 ± 13.97	63.82 ± 13.02	0.37	63.86 ± 13.17	63.65 ± 13.13	0.88	65.55 ± 13.31	62.97 ± 113.00	0.01^*∗*^
Female, *n* (%)	67 (67.68)	299 (40.03)	<0.001^‡^	69 (61.61)	297 (40.46)	<0.001^‡^	116 (50.00)	250 (40.72)	0.02^*∗*^
BMI (kg/m^2^)	25.84 ± 4.33	25.49 ± 3.90	0.41	26.85 ± 4.39	25.33 ± 3.84	<0.001^‡^	25.53 ± 3.63	25.53 ± 4.06	1.00
Waist circumference (cm)	88.96 ± 11.47	89.27 ± 10.32	0.79	93.02 ± 11.67	88.66 ± 10.14	<0.001^‡^	89.17 ± 10.44	89.26 ± 10.47	0.92
Lifestyle behaviors									
Smoking history, yes, *n* (%)	4 (4.04)	37 (4.95)	0.69	6 (5.36)	35 (4.77)	0.79	9 (3.88)	32 (5.21)	0.42
Alcohol consumption, yes, *n* (%)	0 (0)	26 (3.48)	0.06	0 (0)	26 (3.54)	0.04^*∗*^	3 (1.29)	23 (3.75)	0.07
Exercise habits, yes, *n* (%)	73 (73.74)	595 (79.65)	0.17	71 (63.39)	597 (81.34)	<0.001^‡^	174 (75)	494 (80.46)	0.08
Diabetic factors									
FBS (mg/dL)	149.50 ± 58.52	143.70 ± 44.70	0.35	149.10 ± 43.21	143.60 ± 47.00	0.25	145.40 ± 52.87	144.00 ± 43.93	0.71
HbAlc (%)	7.83 ± 1.68	7.66 ± 1.59	0.33	8.00 ± 1.64	7.64 ± 1.60	0.03^*∗*^	7.77 ± 1.74	7.65 ± 1.55	0.36
DMH (year)	9.70 ± 9.19	8.82 ± 7.74	0.36	9.16 ± 8.69	8.88 ± 7.81	0.73	9.76 ± 8.82	8.60 ± 7.54	0.08
OHA use, yes, *n* (%)	92 (92.93)	716 (95.85)	0.19	106 (94.64)	702 (95.64)	0.64	222 (95.69)	586 (95.44)	0.88
Insulin usage, yes, *n* (%)	33 (33.33)	172 (3.03)	0.02^*∗*^	35 (31.25)	170 (23.16)	0.06	70 (30.17)	135 (21.99)	0.01^*∗*^
Lipid profile									
TC (mg/dL)	176.90 ± 40.05	175.20 ± 36.67	0.66	180.60 ± 43.66	174.60 ± 35.91	0.17	174.30 ± 37.22	175.80 ± 37.02	0.58
TG (mg/dL)	156.60 ± 116.60	148.20 ± 163.20	0.52	157.70 ± 122.90	147.90 ± 163.20	0.45	143.00 ± 88.02	151.50 ± 177.90	0.36
HDL (mg/dL)	51.46 ± 13.20	52.46 ± 14.67	0.52	50.66 ± 13.29	52.60 ± 14.67	0.19	51.56 ± 13.25	52.64 ± 14.95	0.31
LDL (mg/dL)	109.40 ± 30.92	106.00 ± 31.30	0.31	111.90 ± 34.19	105.50 ± 30.73	0.05^*∗*^	106.50 ± 32.82	106.30 ± 30.68	0.94
Blood pressure									
SBP (mmHg)	130.60 ± 16.32	131.80 ± 14.39	0.45	132.00 ± 15.09	131.60 ± 14.56	0.79	131.30 ± 14.92	131.70 ± 14.52	0.68
DBP (mmHg)	78.34 ± 10.57	77.51 ± 9.02	0.46	77.11 ± 9.66	77.69 ± 9.14	0.53	76.99 ± 9.77	77.85 ± 8.99	0.23
Kidney function									
Microalbumin (mg/dL)	26.77 ± 79.44	34.15 ± 201.8	0.50	33.01 ± 91.77	33.33 ± 202.50	0.98	29.49 ± 87.91	34.73 ± 218.3	0.62
Cr (mg/dL)	1.14 ± 0.50	1.17 ± 0.57	0.57	1.15 ± 0.48	1.17 ± 0.57	0.64	1.21 ± 0.60	1.15 ± 0.54	0.16
eGFR (mL/min)	68.29 ± 26.60	68.11 ± 21.40	0.95	67.44 ± 23.93	68.24 ± 21.77	0.72	64.40 ± 22.74	69.55 ± 21.65	<0.01^†^
ALB/Cr (*μ*g/mg)	415.90 ± 1821.3	224.30 ± 851.10	0.30	309.20 ± 1140.80	237.10 ± 993.60	0.53	293.30 ± 1235.80	229.10 ± 916.70	0.47
Diabetic retinopathy, *n* (%)	30 (30.30)	306 (40.96)	0.04^*∗*^	35 (31.25)	301 (41.01)	0.05^*∗*^	100 (43.10)	236 (38.44)	0.23

Data were presented as mean ± SD for continuous variable and as number (%) for categorical variable.

*P* values were calculated using the chi-square test for categorical variable and *t*-test for continuous variable. ^*∗*^ *P* < 0.05, ^†^ *P* < 0.01, and ^‡^ *P* < 0.001.

BMI: body mass index; FBS: fasting blood sugar; HbA1c: glycosylated hemoglobin; DMH: duration of diabetes mellitus; OHA: oral hypoglycemic agent; TC: total cholesterol; TG: total triacylglyceride; HDL: high-density lipoprotein; LDL: low-density lipoprotein; SBP: systolic blood pressure; DBP: diastolic blood pressure; GPT: glutamic pyruvic transaminase; Cr: creatinine; eGFR: estimated glomerular filtration rate; and ALB/CR: microalbumin to creatinine ratio.

**Table 2:** Prevalence of albuminuria in patients with T2DM according to body constitution.

BC	Albuminuria	Nonalbuminuria	Total	*P* value
	(*n* = 363)	(*n* = 483)	(*n* = 846)	
	*n* (%)	*n* (%)	*N* (%)	
Yang deficiency				
Yes	56 (56.57)	43 (43.43)	99 (100)	<0.01^†^
No	307 (41.10)	440 (58.90)	747 (100)	
Phlegm stasis				
Yes	63 (56.25)	49 (43.75)	112 (100)	<0.01^†^
No	300 (40.87)	434 (59.13)	734 (100)	
Yin deficiency				
Yes	112 (48.28)	120 (51.72)	232 (100)	0.05
No	251 (40.88)	363 (59.12)	614 (100)	

BC: body constitution; DM: diabetes mellitus. ^†^ *P* < 0.01.

*P* values were calculated using the two-sided chi-square test.

**Table 3:** Unadjusted and adjusted odds ratios and 95% CI for albuminuria in patients with T2DM according to body constitution.

	Albuminuria, OR (95% CI)					
	Yang deficiency		Phlegm stasis		Yin deficiency	
	OR (95% CI)	*P* value	OR (95% CI)	*P* value	OR (95% CI)	*P* value
Model 1	1.87 (1.22–2.85)	0.004^†^	1.86 (1.25–2.78)	0.002^†^	1.35 (1.00–1.83)	0.053
Model 2	2.00 (1.29–3.11)	0.002^†^	1.74 (1.15–2.65)	0.010^*∗*^	1.28 (0.93–1.74)	0.126
Model 3	1.97 (1.26–3.08)	0.003^†^	1.64 (1.07–2.50)	0.023^*∗*^	1.25 (0.91–1.71)	0.170
Model 4	1.99 (1.26–3.14)	0.003^†^	1.66 (1.08–2.56)	0.022^*∗*^	1.28 (0.93–1.76)	0.128
Model 5	1.93 (1.21–3.08)	0.006^†^	1.61 (1.03–2.51)	0.035^*∗*^	1.19 (0.86–1.65)	0.298
Model 6	2.16 (1.31–3.58)	0.003^†^	1.84 (1.15–2.94)	0.011^*∗*^	1.13 (0.80–1.60)	0.485
Model 7	2.26 (1.36–3.75)	0.002^†^	1.92 (1.19–3.08)	0.007^†^	1.13 (0.80–1.60)	0.487

*Model 1* is unadjusted. *Model 2* is additionally adjusted for sociodemographic characteristics. *Model 3* is additionally adjusted for lifestyle behaviors. *Model 4* is additionally adjusted for blood pressure and lipid profile. *Model 5* is additionally adjusted for diabetic factors. *Model 6* is additionally adjusted for eGFR. *Model 7* is additionally adjusted for diabetic retinopathy.

Analysis by logistic regression. ^*∗*^ *P* < 0.05, ^†^ *P* < 0.01.

BC: body constitution, including Yang deficiency, Ying deficiency, and Phlegm stasis. Sociodemographic characteristics: gender, age, BMI, and waist circumference. Lifestyle behaviors: smoke and alcohol drinking history and exercise. Blood pressure: SBP and DBP. Lipid profile: TG, HDL, and LDL. Diabetic factors: FBS, HbA1c, DM duration, oral hypoglycemia agent, and insulin use.

**Table 4:** Adjusted odds ratios and 95% CI for albuminuria in patients with T2DM according to Yang deficiency and Phlegm stasis body constitution.

	Albuminuria	
	OR (95% CI)	*P* value
Non-Yang deficiency and non-Phlegm stasis	1.00	
Yang deficiency	1.59 (0.75–3.37)	0.23
Phlegm stasis	1.30 (0.69–2.45)	0.40
Yang deficiency and Phlegm stasis	3.04 (1.57–5.87)	<0.001^‡^

Non-Yang deficiency and non-Phlegm stasis as reference.

Adjusted for sociodemographic factors, lifestyle, blood pressure, lipid profile, diabetic factors, eGFR, and diabetic retinopathy.

Analysis by logistic regression. ^‡^ *P* < 0.001.

DM: diabetes mellitus. Sociodemographic factors: gender, age, BMI, and waist circumference. Lifestyle: smoking and alcohol drinking history and exercise. Blood pressure: SBP and DBP. Diabetic factors: FBS, HbA1c, DM duration, oral hypoglycemia agent, and insulin use.
